# Amplitude based segmentation of ultrasound echoes for attenuation coefficient estimation

**DOI:** 10.1016/j.ultras.2020.106302

**Published:** 2021-03

**Authors:** John Civale, Jeff Bamber, Emma Harris

**Affiliations:** The Institute of Cancer Research, London SW7 3RP, UK

**Keywords:** Ultrasound, Attenuation, Backscatter, Image segmentation, Breast, B-mode, Radiofrequency

## Abstract

In vivo ultrasound attenuation coefficient measurements are of interest as they can provide insight into tissue pathology. They are also needed so that measurements of the tissue’s frequency dependent ultrasound backscattering coefficient may be corrected for attenuation. In vivo measurements of the attenuation coefficient are challenging because it has to be estimated from the depth dependent decay of backscatter signals that display a large degree of magnitude variation. In this study we describe and evaluate an improved backscatter method to estimate ultrasound attenuation which is tolerant to the presence of some backscatter inhomogeneity. This employs an automated algorithm to segment and remove atypically strong echoes to lessen the potential bias these may introduce on the attenuation coefficient estimates. The benefit of the algorithm was evaluated by measuring the frequency dependent attenuation coefficient of a gelatine phantom containing randomly distributed cellulose scatterers as a homogeneous backscattering component and planar pieces of cooked leek to provide backscattering inhomogeneities. In the phantom the segmentation algorithm was found to improve the accuracy and precision of attenuation coefficient estimates by up to 80% and 90%, respectively. The effect of the algorithm was then measured *in vivo* using 32 radiofrequency B-mode datasets from the breasts of two healthy female volunteers, producing a 5 to 25% reduction in mean attenuation coefficient estimates and a 30 to 50% reduction in standard deviation of attenuation coefficient across different positions within each breast. The results suggest that the segmentation algorithm may improve the accuracy and precision of attenuation coefficient estimates *in vivo*.

## 1 Introduction

Measurement of the ultrasound characteristics of tissues, such as attenuation coefficient, sound speed and backscatter coefficient, can provide insight into tissue state and pathology. There has been considerable interest in estimating ultrasound characteristics in tissues such as liver [1], [2], breast [3], [4], [5], thyroid [6], uterus [7], and muscle [8]. In general, i.e., with the exception of quantitative measurement using ultrasound computed tomography techniques that are mainly applicable to the breast [9], [10], *in vivo* characterisation of these properties is challenging because of the availability only of ultrasound backscatter from tissues, and the difficulty of detecting the non-scattered component after ultrasound has passed through a region of the body. Nevertheless, backscatter methods for measuring these tissue properties have been published [1], [11], [12], and the availability of wideband ultrasound transducers in combination with diagnostic imaging systems that provide access to the radio frequency (RF) data is improving the ability to apply them to solve clinical problems.

The backscatter signal power detected by an ultrasound imaging system, *S*, may be described as arising from the product of a number of depth (*z*) and frequency (*f*) dependent transfer functions, namely the imaging system’s receive transfer characteristic (*T*), diffraction (*D*), the sample’s backscatter coefficient (*B*), the transmitted acoustic input (*I*), and a factor quantifying the effect due to acoustic attenuation (*A*) in the path between the imaging probe and a region of interest [13], [14]:

(1) S(f,z)=T(f,z)D(f,z)B(f)I(f)A(f,z)

The imaging system’s transfer function, *T*, can be understood in terms of the overall response of the system’s receiver to detection of an acoustic stimulus. In practice this component depends on a large range of factors such as: the specific frequency response of the imaging probe being used, receive amplifier gain including time gain compensation, and any signal processing steps including beamforming. Typically, the user may have some control over this parameter in terms of choice of imaging probe, and receive gain settings. The second factor on the right-hand side of Eq. (1) (*D*) refers to effects of diffraction on the transmitted and received ultrasound beam. The third component (*B*) is the backscatter coefficient and is a property of the tissue describing the proportion of the incident ultrasound energy per unit volume scattered back to the imaging probe. This property is largely dependent on the size, density, shape and distribution of scatterers within the tissue, here we assume the backscatter coefficient is spatially homogeneous. A fourth factor (*I*) represents the acoustic output of the probe on transmit and may be controlled to some extent by the user by varying transmit power, focus and apodisation. The final factor in Eq. (1), *A*, describes the proportion of backscatter energy attenuated in the propagation from the probe to the depth in the tissue at which *S* is measured, and back again. The total attenuation is in turn dependent on the cumulative effects of absorption and scattering along the propagation path, which act to reduce the ultrasound intensity. Factor *A* in Eq. (1) may therefore be written:

(2) $$A\left(f,z\right)=e^{-2\int_{0}^{z}\alpha \left(f,\gamma \right)d\gamma }$$

where *α* is the frequency and spatially dependent intensity attenuation coefficient in units of Nepers per cm, *z* is the depth position, and *γ* is the path integration variable.

To quantify attenuation, the difference in power spectra between two or more depth regions is required (spectral log difference method) [15]. These spectra will, however, be dependent on all the factors in Eq. (1) and not attenuation alone. Some of these factors can be controlled to some extent by the user, for example effects due to diffraction (*D*) and instrumentation (*T, I*) can be minimised or corrected by diffraction filtering [11], [16], or by using echo data obtained from a reference phantom with known backscatter and attenuation properties [14].

Different tissue types will inevitably exhibit different scattering properties, and the interface between tissue layers may give rise to specular reflections of significantly greater magnitude than the surrounding tissue backscatter. Backscatter coefficient (*B*) inhomogeneity can therefore provide a source of bias in BAE measurements. Even within a single organ or tissue, substantial backscatter heterogeneity may occur, the breast being an important example. Furthermore, the inherent variance in the backscatter signal arising from speckle [17] leads to a lack of precision in BAE estimates that can only be reduced by a large amount of spatial averaging [18].

Here, our interest is in using backscattered ultrasound to measure the frequency dependence of the attenuation coefficient, a potentially useful tissue characteristic in its own right but one that is also needed to apply an attenuation correction for accurate measurement of the frequency dependent backscatter coefficient of tissue. This is particularly true for regions of tissue at depth where the attenuation of overlying tissues can significantly alter the magnitude and spectrum of detected backscatter ultrasound signals. Since the detected backscatter signals depend on both backscatter and attenuation properties, methods often rely on assumptions regarding the homogeneity of attenuation and backscatter properties. A common approach is to image a reference phantom [14] with well characterised backscatter and attenuation properties. The frequency dependent sample to reference backscattered echo power ratio is computed, and the attenuation and backscatter coefficient of the sample can then be derived from these data by performing a linear regression on the log of these ratio as a function of depth. Other authors have considered non-parametric approaches to measure attenuation properties, for example the centroid frequency shift [19], or related hybrid methods [20] which do not rely on the amplitude of the detected echoes. Methods such as these were found to be less susceptible to backscatter inhomogeneity, however they rely on other assumptions which are usually invalid, such as for example a Gaussian shaped pulse spectrum or homogeneity of frequency dependence. Nam *et al* [21] considered the problem of spatial inhomogeneity in terms of a phantom with layers of differing attenuation and backscatter properties. They proposed a reference phantom approach with the addition of a constrained least squares fitting method aimed at determining the attenuation frequency dependence. They found their least squares method was less susceptible to errors in the presence of backscatter inhomogeneity when compared to the reference phantom method alone. More recent developments include the use of spatial compounding using steered beams [22], or by mechanically rotating the imaging probe [23], to generate a larger number of independent measurements and thus reduce error due to speckle. The envelope of RF signals has also been considered as a means to detect deviations from the Rayleigh distribution that is usually assumed for randomly spaced large scatterer densities [24]. These authors showed an increase in the variance of attenuation estimates when the backscatter signals did not conform to Rayleigh distribution statistics. Deeba et al [25] quantified the change in signal to noise ratio of the RF envelope and variation in the ultrasound pulse bandwidth to calculate attenuation estimates only from regions of tissue with homogeneous backscattering properties. This method demonstrated a reduction in the variance of attenuation coefficient estimates, but requires discretisation of the image data in order to determine the necessary RF envelope statistics. Other authors have considered data regularization methods, with the aim of generating quantitative parametric images of attenuation [26], [27], [28] and backscatter properties [29] with the potential to greatly enhance medical diagnosis. Such methods rely on the minimisation of cost functions evaluated using total variation regularization [26], [27] and have demonstrated significant improvement over simple log spectral methods in detecting localised changes in attenuation properties. These methods however also rely on assumptions regarding the frequency dependence of attenuation coefficients, and the medium is assumed to be piecewise homogeneous. Furthermore, an optimisation of regularization parameters is also required which may be dependent on anatomy and imaging equipment.

In this paper we aim to discover whether pre-processing can be applied to measured backscatter data to improve accuracy and precision of attenuation coefficient estimation by selecting only backscatter data that conforms to the assumptions of Eq. (1). We propose to achieve this by applying an iterative intensity-based segmentation algorithm. We test our algorithm on data obtained from a specially constructed test phantom containing randomly distributed scatterers with a number of embedded planar reflectors Our method therefore represents a development and refinement of earlier work by Laugier [30] who considered the effect of strong reflectors in backscatter attenuation estimation. We conclude by reporting the results of testing our algorithm on clinical data from human breast scans. Our method therefore differs from those of previous studies in two ways. Firstly, we obtain frequency dependent attenuation coefficients averaged over all the available echo data without attempting to produce maps of attenuation properties. Secondly, our segmentation algorithm offers an alternative to methods based on quantifying statistics of the RF envelope distributions [25], our segmentation method can be performed directly on the RF data without the need to define blocks of data over which to quantify parameters. The main assumption of interest is that the backscattering coefficient is homogeneous. A further important assumption, that the backscattering is associated with only one type of ultrasound scattering structure (i.e., level of coherence, frequency dependence, etc.), is the subject of future work.

## 2 Materials and methods

### 2.1 Overall approach

Our approach is to use a short time multi-narrowband log-spectral approach applied to the backscatter echo data. An automated algorithm is implemented on the radiofrequency (RF) data where the aim is to eliminate the echoes corresponding to hyperechoic features which we refer to as backscatter outliers. The algorithm can be considered as an extension of previous work by Laugier *et al* [30] who found attenuation coefficient estimates to be sensitive to the source of hyperechoic structures in otherwise homogeneous backscattering material. Our proposed method extends this by determining a depth dependent intensity thresholding function. Backscatter outliers are detected using this threshold function, and the corresponding RF data is not included in subsequent attenuation estimation. The algorithm was tested on a specifically constructed proof-of-concept test phantom with embedded hyperechoic backscatter outliers. Phantom backscatter attenuation estimates were compared with independent transmission measurement results. Finally, the segmentation algorithm was applied to RF echo data from B-mode images of the female breast obtained in the clinic. Breast tissue is inhomogeneous, with a combination of fatty tissue, ducts and fibroglandular tissue. This inhomogeneity manifests as backscatter inhomogeneity and therefore presents an opportunity to test the segmentation of backscatter outliers. Previous measurements on breast tissue have demonstrated how the attenuation is dependent on tissue morphology and pathology in excised tissue samples [3], and *in vivo* [5]. In this work we restrict ourselves to testing the algorithm on data obtained from healthy volunteers to assess its potential.

### 2.2 Ultrasound imaging system

A Verasonics V1 system (Verasonics Inc., WA, USA) was used with a 128 element L7-4 (Philips Medical Systems, The Netherlands) linear array probe to capture ultrasound echo data. The V1 system allowed full control over pulse transmission events and allowed capture of the channel level raw RF data. A line-by-line B-mode imaging sequence was designed with the following criteria: transmit aperture size of 27 elements, transmit frequency of 5 MHz (nominal probe centre frequency with a usable bandwidth of 3–6.5 MHz), with a single transmit focal point set to a depth of 50 mm and an excitation voltage amplitude set to 5.3 V. A flat time gain compensation profile was applied and maximum imaging depth was set to 60 mm. To maintain overall control over the B-mode image formation an in-house beamforming algorithm was developed for offline data processing and image formation in Matlab (Mathworks ®, MA, USA). A fixed receive aperture of 27 elements was maintained, identical to the transmit aperture, resulting in a final image consisting of 102 beamformed RF lines. A dynamic focusing approach was taken whereby the receive delays applied to the channel RF data were dynamically changed with depth, eliminating the need to specify receive focal depths. A sound speed of 1540 m/s was used for image reconstruction.

### 2.3 Test phantom

**Fig. 1:**
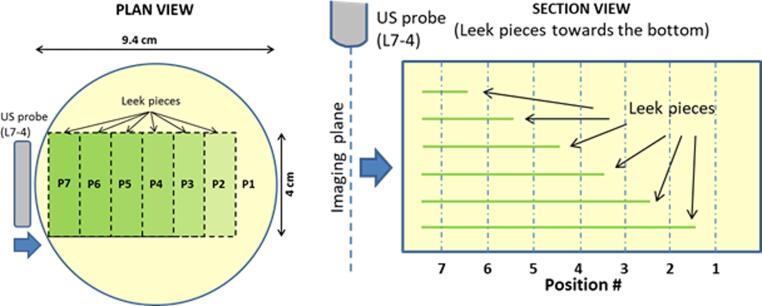
Plan (left) and section (right) views of the phantom. Imaging positions are indicated with respect to the arrangement of the rectangular leek pieces. Orientation A is defined as imaging from the top with the larger leek pieces towards the bottom (as shown here), and orientation B is defined as imaging the phantom after rotating through 180° so that the larger leek pieces are nearer the top.

The test phantom was developed using water and gelatine from porcine skin (Type A, 175 bloom, Sigma-Aldrich, UK). The gelatine content consisted of 10% of the phantom by mass, and contained cellulose powder scatterers (1% mass). The phantom was set a layer at a time in a cylindrical mould with a diameter of 8.5 cm and a depth of 5 cm. Planar sections of leek were obtained by performing parallel transaxial cuts at 4 cm spacing producing a cylindrical section consisting of concentric circular layers. The outer layers were separated and by softened by cooking in boiling water for five minutes, and subsequently cut to the required size and placed flat on each layer of gel as shown in Fig. 1. This procedure allowed construction of a phantom with cross sections containing no leek pieces (position 1) at one end through to the opposite end (position 7) where the leek pieces were placed uniformly throughout the depth range. The leek pieces provided a hyperechoic signal source compared to the surrounding speckle pattern produced by the cellulose scatterers. The cylindrical mould was capped at both ends using a Melinex® membrane (thickness 19 μm) providing a water tight acoustic window suitable for ultrasound imaging from both ends, thus providing measurements in two orientations: orientation A where the leek pieces were placed towards the bottom of the phantom, and orientation B with the phantom rotated through 180° so that the leek pieces were positioned towards the top of the phantom.

### 2.4 Backscatter attenuation estimation

The backscatter power *S*, detected by the imaging probe, can be considered the result of a series of frequency *f* and depth *z* dependent transfer functions [13]. By combining Eqs. (1), (2) above we have:

(3) $$S\left(f,z\right)=T\left(f,z\right)D\left(f,z\right)B\left(f\right){I(f,z)e}^{-2\alpha \left(f\right)z}$$

where a spatially homogeneous attenuation coefficient *α* has been assumed. It is possible however to combine *T*, *D, B and I* into a single parameter *H,* if backscatter coefficient homogeneity is also assumed we have:

(4) $$H\left(f,z\right)=T\left(f,z\right)B\left(f\right)D\left(f,z\right)I\left(f,z\right)$$

Henceforth we shall refer to *H* as the diffraction correction function that needs to be characterised to proceed with attenuation estimation. It must be emphasised that the term chosen here reflects the fundamental physical effect that must be corrected, in this context it also includes the imaging system’s response (*T*) which covers properties such as the imaging system’s usable bandwidth, filtering, time gain compensation characteristics etc., as well as the backscatter properties (*B*) of a calibration or reference material, as well as the input function *I*. The detected power *S* at a given frequency as a function of depth *z* is then only dependent on *H* and the attenuation properties of the sample

(5) $$S\left(f,z\right)=H\left(f,z\right)e^{-2\alpha \left(f\right)z}$$

If the form of *H* can be quantified, *S* can be normalised by *H* obtaining a diffraction corrected parameter *S_d_* that follows an exponential decay curve

(6) $$S_{d}\left(f,z\right)=\frac{S\left(f,z\right)}{H\left(f,z\right)}\propto e^{-2\alpha \left(f\right)z}$$

The attenuation coefficient can therefore be estimated by measuring the gradient of a straight line fitted to the log of *S_d_* versus depth *z* at each frequency*.*

The diffraction correction function *H* to be used for backscatter attenuation estimation was characterised with the Verasonics V1 system configured with identical settings to those used during BAE measurements on the test phantom. The diffraction correction function *H* was characterised by imaging homogeneous sections of the test phantom. Frequency and depth dependent values of *S* were obtained by defining region of interest (ROI) windows which consist of short segments of RF data centred at increasing values of *z*. The RF data was segmented into ROI windows with 50% overlap in the axial direction, with width covering all of the 102 lines RF data lines. Three ROI window lengths were used equivalent to 30, 18 and 12 cycles at the nominal centre frequency (5 MHz), these were equivalent to 120, 72 and 48 RF samples respectively, or times of 6, 3.6 and 2.4 μs, assuming a sound speed of 1540 m/s. Hanning windows were applied to the backscatter data segments and the power spectrum was obtained by FFT. These spectra were averaged over all of the 102 A–lines to produce reference detected backscatter power *S_r_*, providing *H* after attenuation correction (see Eqs. (1), (2)) where *α* is in units of intensity Np/cm and was measured using an independent transmission measurement method:

(7) $$H\left(f,z\right)=S_{r}\left(f,z\right)e^{2\alpha \left(f\right)z}$$

This procedure was repeated for a total of 6 independent scan planes in the phantom, the corresponding diffraction characterisations were averaged providing a final *H* data set with improved signal to noise ratio.

Attenuation coefficients were then determined by imaging at the different positions within the test phantom, acquiring the RF data from the test phantom and segmenting the data according to the ROI widths and positions that were set when characterising the diffraction correction data set *H*. In this step the depth dependent values of *H* were normalised to a value of 1 at the first (shallow) depth position for all frequencies.

The diffraction corrected data at each frequency component were converted to dB and a linear fit was applied to these data when plotted against the depth (*z*) using the method of least squares providing a slope (*b*) and zero-depth offset (*a*). The gradient of the line of best fit was halved to account for the pulse echo nature of the signal to obtain attenuation coefficient estimates in dB/cm, and a mean normalised residual *y* in dB was calculated from the fitted parameters:

(8) $$y\left(f\right)=\sqrt{\frac{1}{N}\sum_{i}^{N}{\left(10\log_{10}\left(S_{i}\left(f,z\right)\right)-a\left(f\right)-b\left(f\right)z_{i}\right)}^{2}}$$

A power law of the form:

(9) $$\alpha \left(f\right)=\beta f^{n}$$

was applied to the attenuation coefficient vs frequency results. The fits were applied over the usable frequency range of the L7-4 imaging probe (3.0–6.5 MHz). A second mean normalised residual was obtained from the attenuation coefficient data in units of dB/cm quantifying the deviation of the attenuation coefficient measurements from those obtained using a reference transmission measurement.

## 3 Reference transmission attenuation measurements

Independent transmission attenuation measurements were possible on the test phantom due to the acoustic windows present on both sides. This arrangement was favourable as it allowed an independent evaluation of the attenuation properties of the phantom against which to compare the backscatter based estimates. A pulse–echo approach was used, a planar Perspex™ reflector was placed distal to the phantom with a short gap (1–2 cm) separating it from the back window of the phantom. The imaging sequence was modified so that the transmit focus was placed at the surface of the planar reflector after this had been carefully aligned normal to the sound axis. RF echo data were obtained for all positions of the phantom. The phantom was then removed and the distance between the imaging probe and reflector adjusted to reposition the echo at the same apparent location on the image as during the phantom measurements. This adjustment was made to correct for any diffraction errors due to differences in the sound speed between the phantom and the water. A water path only set of RF data was thus obtained, and the attenuation frequency dependence was calculated:

(10) $$\alpha \left(f\right)=\frac{10\log_{10}\left(\frac{P_{r\left(f\right)}}{P_{m}\left(f\right)}\right)}{2x}$$

where *α* is the attenuation coefficient in dB/cm, *P_r_* and *P_m_* are the mean power spectra of the reference and attenuated echoes respectively, and *x* is the phantom thickness (5.0 ± 0.1 cm). A correction for transmission losses at the water/gel interfaces was not considered.

### 3.1 Clinical data

The segmentation algorithm was tested on B-mode image RF data of the breast provided by two healthy female volunteers (ages 25 and 59 respectively) scanned as a part of a separate study (clinical trial NCTO2388230). In vivo data was obtained with approval from the Health Research Authority (HRA) and Research Ethics Committee (REC), informed consent was obtained from the volunteers. The data sets were selected on the basis of providing sufficient breast tissue to fill the imaging depth range (6 cm). Each volunteer provided a total of 16B-mode image data sets, in two probe orientations (radial and anti-radial) in each quadrant, for each breast. Attenuation estimates were computed firstly without the automated segmentation algorithm, and subsequently with the segmentation algorithm with three backscatter target residual range (TRR) settings (see below). B-mode image data obtained with the Verasonics V1 system using a standard B-mode imaging sequence similar to the one described above for the test phantom measurements. The diffraction correction for this sequence was characterised separately in the laboratory using a gelatine phantom loaded with high density poly ethylene (HDPE) scatterers (mean diameter 119 μm). The technique used to characterize the diffraction field *H* in this case was that of an axial beam translation with water as the intervening path between the probe and the phantom (Fink and Cardoso 1984). The phantom was moved in steps of 2.5 mm axially away from the probe, and for each position an image was obtained. In each image an ROI window was set with the leading edge a short distance (<2mm) inside the phantom. This procedure allowed characterisation of the diffraction field *H* over a number of axial positions by determining the average power spectrum as described previously. The main difference with the method described above is that an attenuation correction was not necessary as the ROI windows were all set at constant and shallow depth in the phantom.

### 3.2 Intensity thresholding and segmentation

The aim of intensity based image segmentation was to remove backscatter outliers in the computation of attenuation estimates in order to improve accuracy. The diffraction field data *H* and an initial estimate of the attenuation coefficient frequency slope (*β,* in Npcm/MHz) were used to determine a depth dependent amplitude threshold function which represents the mean backscatter amplitude that might be expected from a homogeneous sample. To calculate this threshold function *F*, the diffraction function spectral power components, *f* (MHz), were attenuation corrected and summed at each depth (*z*) position, and square rooted to convert to amplitude:

(11) $$F\left(z\right)=s2\sqrt{\sum_{i=1}^{N}H\left(f_{i},z\right)e^{-2\beta f_{i}z}}$$

**Fig. 2:**
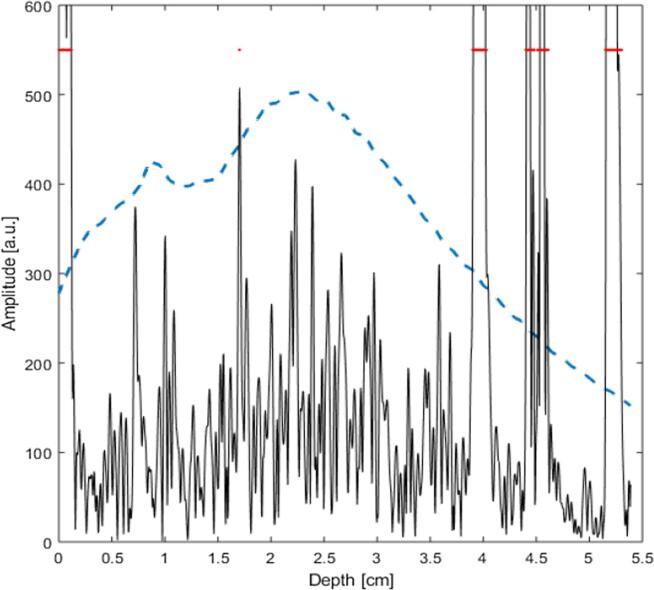
Segmentation example showing an A–line (black line) obtained from the phantom at imaging position 3 (see Fig. 1) with two leek pieces placed at the distal region. The outline of the threshold function *T* (dashed line) is included. Locations where the A line RF data envelope exceeds the threshold function are consistent with the front and back surfaces of the phantom (0.1 and 5.3 cm respectively), and the two leek pieces (at 4.0 and 4.5 cm depth).

A scaling factor *s* is included above, and was necessary to act as a means to control the relative amplitude of the threshold function. In a homogeneous tissue with a large number of randomly distributed point scatterers the scaling factor should be set in a way to preserve the expected backscatter speckle distribution. For an inhomogeneous material containing backscatter outliers - for example strongly reflecting structures or regions with backscatter coefficient much larger than their surroundings - the ideal scaling factor setting is one which effectively places the high amplitude echoes from the backscatter outliers above the threshold function. A suitable choice of scaling factor will therefore segment out only backscatter outliers, leaving relatively homogeneous background echoes for attenuation estimation satisfying the requirements for Eqs. (4), (5). The threshold function was applied to the Hilbert transform of the RF data A lines, an example is shown in Fig. 2.

**Fig. 3:**
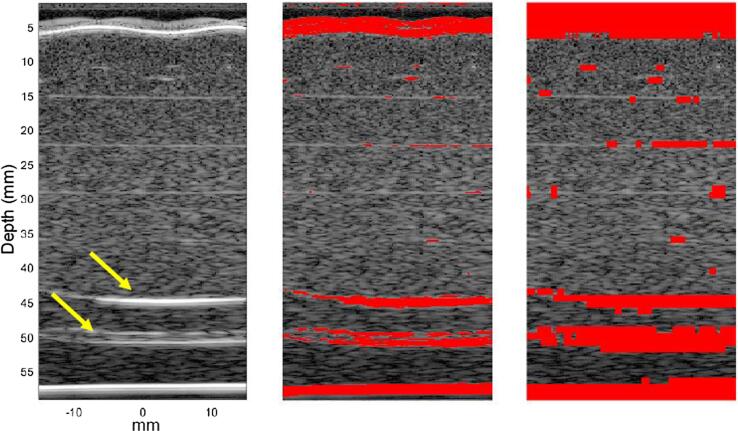
Example of the B-mode image (left) of the phantom with leek pieces indicated (yellow arrows). The result of applying the threshold function is shown (centre) with the segmented areas highlighted in red. The segmented image is used to determine segmentation of ROI measurement windows, resulting in the final image (right) where the highlighted regions indicate areas which do not contribute towards attenuation estimation.

During backscatter attenuation estimation the ROI window positions were compared to the image mask, any ROI window data segments where the mask was found to overlap the central half of the segment were excluded from attenuation estimation. An example segmentation mask and its effect on ROI window data segmentation is given in Fig. 3.

### 3.3 Automated image segmentation

The process of determining an appropriate scaling value for the segmentation process was considered in terms of the statistical distribution of backscatter measurements. Analysis of the Rayleigh distribution which applies to fully developed ultrasound backscatter speckle in a homogeneous medium, shows a constant ratio between the standard deviation and mean value equal to 0.524 irrespective of the mean value, corresponding to approximately 5.6 dB. When considering the backscatter from a region of tissue there are therefore four sources of variance: the first is due to the speckle nature of the signal, the second is due to variation in backscatter coefficient, the third is due to attenuation in the path, and finally a fourth source encompassing other factors such as for example diffraction, or errors in applying a suitable diffraction correction. The exponential decay fitting process was performed by converting the backscatter data to dB, and applying a linear fit as a function of depth. This process also yielded the normalised residual, a measure of the mean deviation of data from the line of best fit. The residual value was therefore taken as the quality metric on which to base the feedback loop of the automated segmentation algorithm.

**Fig. 4:**
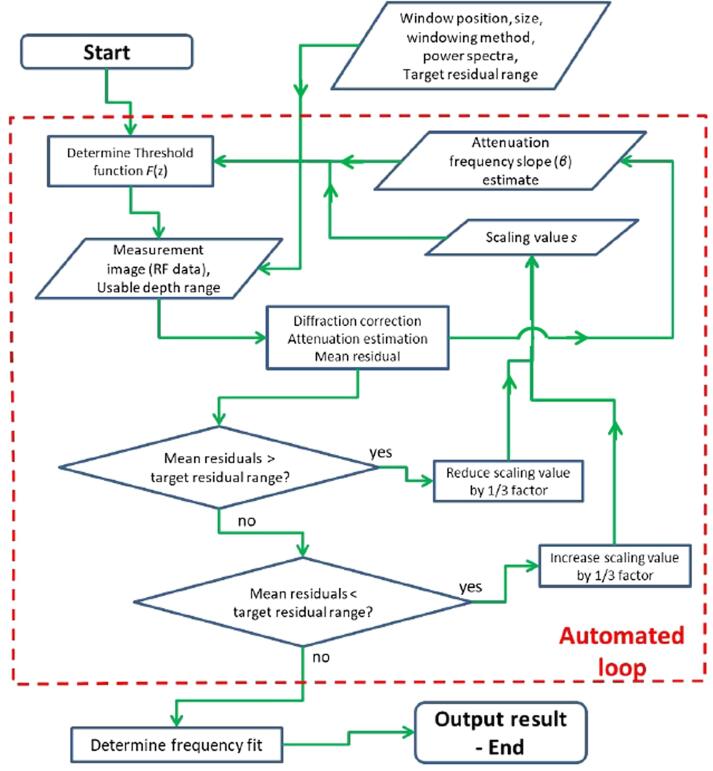
Automated segmentation algorithm flow-chart.

The algorithm was based on a feedback loop with the normalised residual as the test parameter, this is illustrated in a flow chart (Fig. 4). A mean TRR (i.e. 5.6–6 dB) was defined a priori as an exit clause from the loop. The algorithm began by calculating the threshold function scaling parameter large enough to retain all backscatter data for attenuation estimation. The average residual from the fitting process across frequencies was determined, if this parameter was found to exceed the mean TRR the scaling parameter *s* was reduced by a factor of 1/3. The algorithm proceeded iteratively in this manner recomputing the attenuation frequency slope *β* and reducing the scaling parameter and associated threshold function at each step until the mean residual was found to be within the TRR. If during the iteration the mean residual was found to be smaller than the target range, the scaling factor value was increased accordingly. There were two eventual outcomes: the first being the algorithm eventually exits the feedback loop and produces a result; or the feedback loop continued eliminating data until there was insufficient data for attenuation estimation, leading to an error. The latter case could occur due to a backscatter signal probability density function with a lower than expected signal-to-noise ratio, or due to spatial variations in tissue backscatter properties that cannot be filtered out by the algorithm. In these cases the TRR setting would have to be increased in order for the algorithm to produce a result.

For the test phantom measurements, a TRR of 5.6–6 dB was used, a range appropriate for a Rayleigh distributed backscatter speckle distribution. For the clinical data where the assumption of Rayleigh distributed backscatter echoes could not be made, and where spatial distribution of backscatter inhomogeneity was different to the test phantom, the TRR was evaluated over a number of ranges (5.6–6.4 dB, 6.0–6.8 dB, and 6.4–7.2 dB) in order to evaluate settings which might be most useful in a clinical setting.

## 4 Results

### 4.1 Phantom

**Fig. 5:**
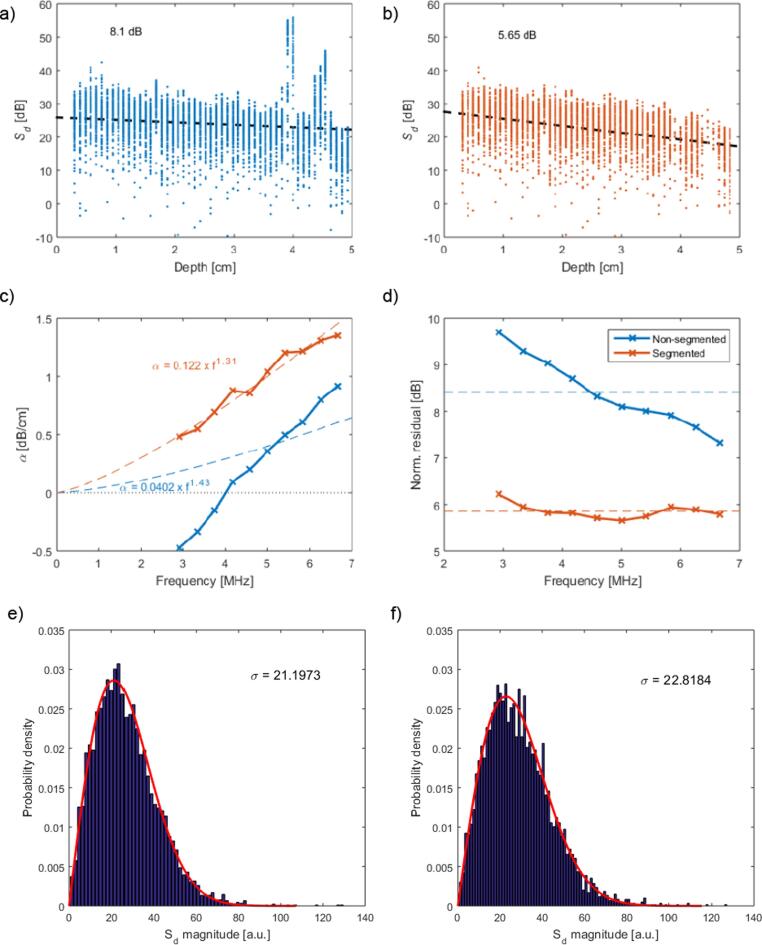
The 5 MHz components of S_d_ prior to (a), and after (b) segmentation are plotted versus depth, the linear fit (dashed line) used to determine the attenuation coefficient is included together with the normalised residual.. The frequency dependent attenuation coefficients with (red) and without (blue) segmentation are plotted (c) together with their respective power law fits. The respective normalised residuals (d) show a consistent reduction at all frequencies, the dashed lines indicate the mean. Probability density functions were computed from attenuation corrected S_d_ values for position 1 (e), and position 3 of the phantom after segmentation (f). Rayleigh distributions were fitted to the experimental data (red curves).

One B-mode image set of RF data was obtained for each position, and two orientations of the test phantom. The data were processed using the 3 different ROI windows, defined when characterising the diffraction function. Data were processed without segmentation first, and then with the automated segmentation algorithm applied. An example of the data processing steps is given in Fig. 5 Diffraction corrected backscatter versus depth data at 5 MHz is plotted in Fig. 5a and 5b, illustrating the removal of the higher magnitude echoes from the leek pieces and the resulting change in the attenuation coefficient from the linear fit.

**Fig. 6:**
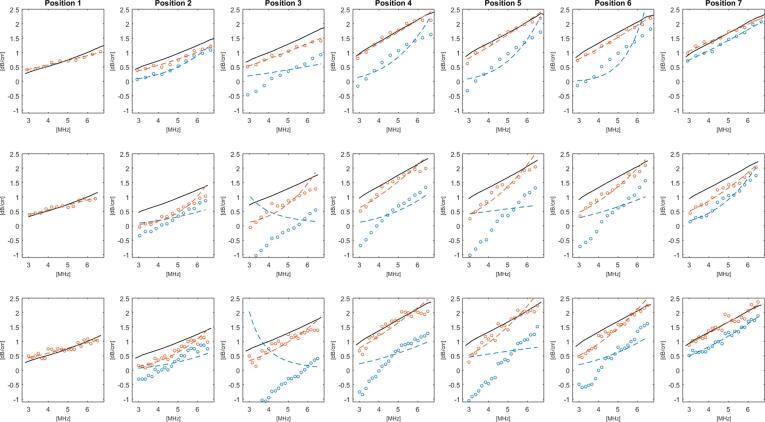
Attenuation coefficients versus frequency plots for the phantom in orientation A, i.e., with the larger leek pieces towards the bottom, for positions 1–7 (columns) and ROI window size: 2.4 μs (top row), 3.6 μs (centre row) and 6.0 μs (bottom row). The black lines indicate the results of the reference transmission attenuation measurement, red and blue markers represent the backscatter attenuation coefficient estimations with and without segmentation, respectively, the dashed lines represent the power law fits applied to the backscatter attenuation measurements.

**Fig. 7:**
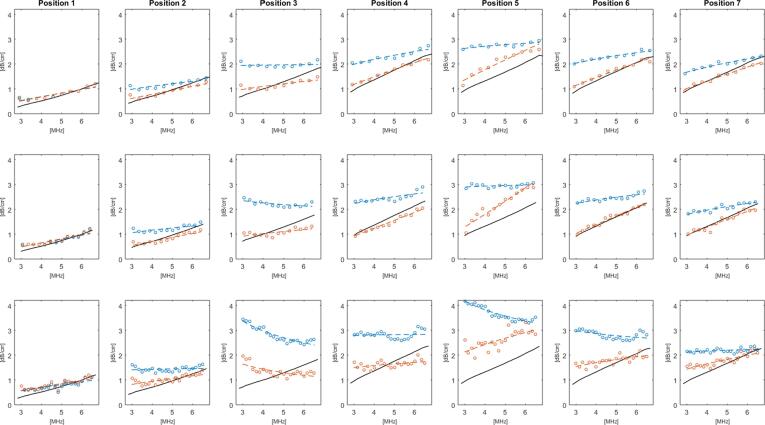
Attenuation coefficients vs frequency plots for the phantom in orientation B, i.e., with the larger leek pieces at the top, for positions 1–7 (columns) and ROI window size: 2.4 μs (top row), 3.6 μs (centre row) and 6.0 μs (bottom row). The black lines indicated the results of the reference transmission attenuation measurements, red and blue markers represent the results he backscatter attenuation coefficient estimations with and without segmentation, respectively, the dashed line represent the power law fit applied to the backscatter attenuation measurements.

The frequency dependence (Fig. 5c) and mean residuals (Fig. 5d) show the effect of segmentation. Finally examples of the probability density functions for attenuation corrected S_d_ data are shown in Fig. 5e and 5f for position 1, and position 3 after segmentation These probability density functions were found to provide a good fit to a Rayleigh distribution, from which a TRR range of 5.6 to 6 dB was chosen. The attenuation coefficient results are plotted in Fig. 6 for each ROI window size and scan position for leek pieces positioned at the bottom (orientation A). Reference attenuation measurements are included in the plots allowing a comparison of the relative accuracy of the data processing methods. When there was an inhomogeneous distribution of the leek pieces, such as for example in positions 3 to 5, backscatter attenuation estimates without segmentation provided very low values when the leek pieces were at the bottom. When segmentation was applied, backscatter attenuation coefficient estimates were much closer to the reference values. When the phantom was rotated (orientation B) so that the leek pieces were arranged from the top, at positions 3 to 5, very high backscatter attenuation coefficient estimates were obtained without segmentation when compared to the reference values, as shown in Fig. 7. When the segmentation algorithm was applied the attenuation coefficient estimates were reduced and were found to be closer to the reference attenuations coefficient values.

Mean deviations in backscatter attenuation coefficient estimates from the reference values are summarised in Table 1, showing the effect of applying the segmentation was to significantly reduce the errors in backscatter attenuation estimates by as much as 80%. The mean error of the segmentation algorithm results were consistently within 0.2 and 0.5 dB/cm of the reference value for the orientations A and B of the leek pieces, respectively, whereas the equivalent estimates without using the algorithm were found to be in excess of 0.8 dB/cm with a peak value of 2 dB/cm. For the phantom positions with fewer leek pieces (positions 2 and 3), or with more pieces where these were distributed throughout (positions 6 and 7) the differences between the processing methods were less marked. A paired *t*-test applied to the attenuation coefficient data showed statistically significant differences (p < 0.05) between segmented and non-segmented measurements for both phantom orientations, ROI window sizes, at all positions containing leek pieces (positions 2–7). Overall, the 12 cycle ROI window size was found to give better agreement with the reference measurements.

**Table 1:** Mean attenuation coefficient deviation from the reference measurement (dB/cm) for the test phantom in terms of imaging position and window size. Values are reported with and without use of the segmentation algorithm for orientation A (leek pieces towards the bottom) and orientation B (leek pieces towards the top). Values in bold font represent lower residual when comparing between segmented and non-segmented results.

	**Mean deviation from reference - Orientation A (dB/cm)**						**Mean deviation from reference - Orientation B (dB/cm)**					
**Position**	**30 cycles**		**18 cycles**		**12 cycles**		**Orientation B 30 cycles**		**Orientation B 18 cycles**		**Orientation B 12 cycles**	
	**Non-Seg.**	**Seg.**	**Non-Seg.**	**Seg.**	**Non-Seg.**	**Seg.**	**Non-Seg.**	**Seg.**	**Non-Seg.**	**Seg.**	**Non-Seg.**	**Seg.**
**1**	0.11	0.11	0.09	0.09	0.11	0.11	0.17	0.18	0.11	0.11	0.14	0.13
**2**	0.66	**0.32**	0.68	**0.45**	0.41	**0.19**	0.59	**0.21**	0.37	**0.13**	0.32	**0.13**
**3**	1.77	**0.31**	1.53	**0.51**	1.00	**0.26**	1.69	**0.50**	1.07	**0.26**	0.80	**0.24**
**4**	1.29	**0.20**	1.23	**0.26**	0.81	**0.11**	1.24	**0.36**	0.87	**0.19**	0.69	**0.13**
**5**	1.43	**0.27**	1.42	**0.36**	0.85	**0.12**	2.17	**1.05**	1.41	**0.63**	1.20	**0.49**
**6**	1.18	**0.28**	1.23	**0.34**	0.81	**0.15**	1.31	**0.34**	0.90	**0.08**	0.75	**0.14**
**7**	0.47	**0.13**	0.71	**0.37**	0.24	**0.08**	0.70	**0.29**	0.54	**0.13**	0.47	**0.13**
**Mean**	0.99	0.23	0.99	0.34	0.60	0.15	1.12	0.42	0.75	0.22	0.63	0.20

The normalised residual obtained in applying a power law fit to the frequency dependence of the attenuation coefficients (Table 2) confirmed an improved fit when the segmentation algorithm was applied for phantom orientation A, whereas for phantom orientation B there was no significant difference. This difference can be understood in terms of the nature of the attenuation coefficients which were significantly under-estimated without segmentation in orientation A, giving very poor power law fits (Fig. 6). For position 1, orientation A, the segmentation algorithm did not need to remove any data to achieve the mean TRR, giving identical results to data processing without segmentation. With the phantom rotated to orientation B, the algorithm segmented a few locations at the interface of the gel layers, this resulted in a significant, albeit minimal difference in the attenuation measurements when using the 30 cycle ROI window size.

**Table 2:** Mean attenuation coefficient frequency dependence fit residual (dB/cm) for the test phantom in terms of imaging position and window size. Values are reported with and without use of the segmentation algorithm for orientation A (leek pieces towards the bottom) and orientation B (leek pieces towards the top). Values in bold font represent lower residual when comparing between segmented and non-segmented results.

	**Mean frequency fit residual - Orientation A (dB/cm)**						**Mean frequency fit residual - Orientation B (dB/cm)**					
**Position**	**30 cycles**		**18 cycles**		**12 cycles**		**30 cycles**		**18 cycles**		**12 cycles**	
	**Non-Seg.**	**Seg.**	**Non-Seg.**	**Seg.**	**Non-Seg.**	**Seg.**	**Non-Seg.**	**Seg.**	**Non-Seg.**	**Seg.**	**Non-Seg.**	**Seg.**
**1**	0.089	0.089	0.055	0.055	0.038	0.038	0.110	**0.099**	0.085	**0.067**	0.081	**0.075**
**2**	0.261	**0.132**	0.244	**0.126**	0.068	**0.059**	**0.094**	0.108	0.082	**0.076**	0.077	**0.070**
**3**	1.539	**0.170**	1.052	**0.237**	0.341	**0.043**	**0.108**	0.156	**0.085**	0.097	**0.093**	0.095
**4**	0.476	**0.213**	0.363	**0.182**	0.282	**0.133**	0.148	**0.123**	0.116	**0.060**	0.078	**0.040**
**5**	0.791	**0.223**	0.738	**0.210**	0.264	**0.123**	**0.098**	0.239	**0.067**	0.142	**0.057**	0.133
**6**	0.459	**0.132**	0.530	**0.121**	0.538	**0.052**	0.130	**0.115**	0.070	**0.064**	**0.045**	0.061
**7**	**0.077**	0.129	0.130	**0.096**	**0.045**	0.054	**0.071**	0.114	**0.067**	0.105	**0.048**	0.051
**Mean**	0.527	0.155	0.444	0.147	0.225	0.072	0.108	0.136	0.082	0.087	0.068	0.075

A paired *t*-test performed on the attenuation coefficient data showed significant differences (p value < 0.05) in the results between different ROI window sizes. Though the use of the segmentation algorithm often gave results in good agreement with the reference measurements, there were some exceptions. For example in orientation A, positions 2 and 3 consistently produced under-estimates of the attenuation coefficients when compared to the reference measurement. In orientation B, the segmentation algorithm tended towards a flatter frequency response, particularly for the larger ROI window size. Inspection of the data suggests this is primarily driven by attenuation coefficient over-estimates towards the lower end of the usable frequency range, however a satisfactory explanation for this observation has not been identified. The large over-estimate of attenuation coefficients at position 5 with the large ROI window size was found to represent a less than ideal implementation of the segmentation algorithm where a very large amount (>80%) of the available RF data was removed, a quantity in excess of all the other positions including position 7 where leek pieces were placed throughout the phantom.

**Fig. 8:**
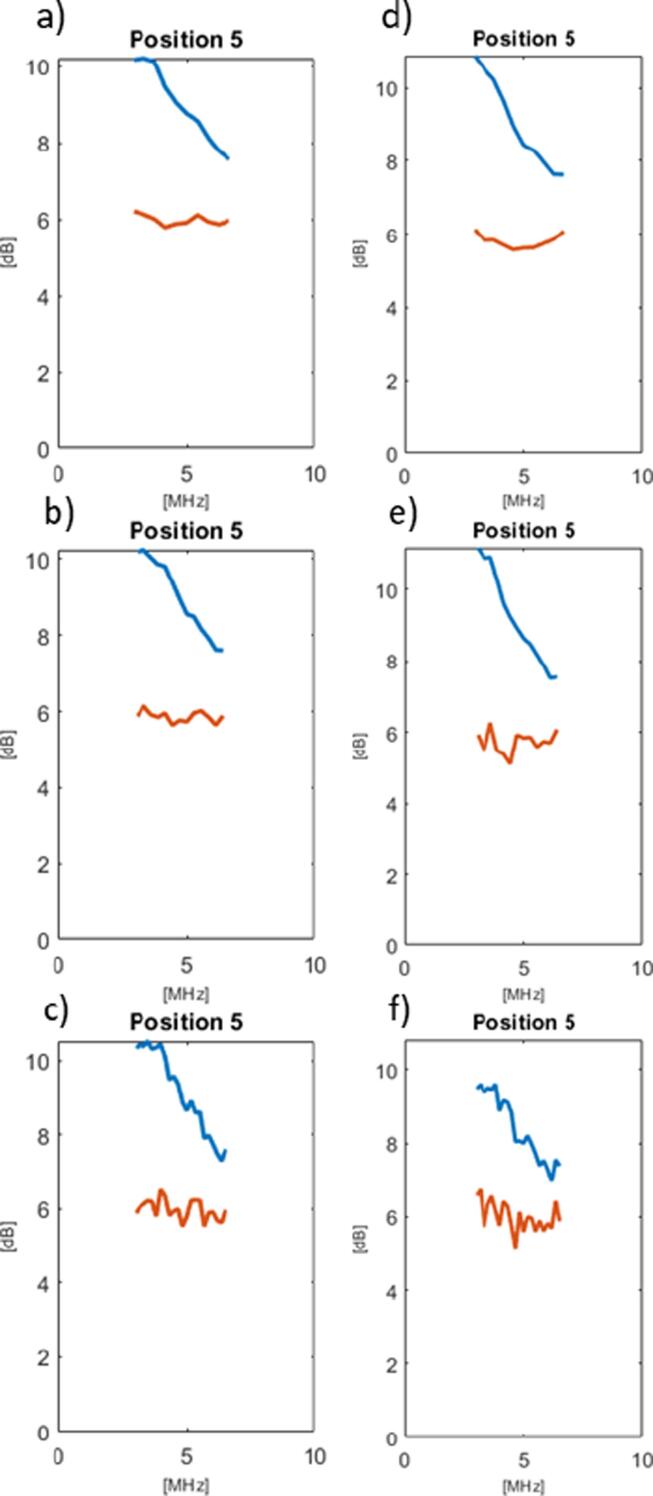
Plots of mean residuals during the attenuation coefficient estimation fitting as a function of frequency, for the phantom scanned at position 5. Plots show results for orientation A (a, b and c) and orientation B (d, e and f), for ROI window sizes 2.4 μs, 3.6 μs and 6.0 μs respectively. The red and blue lines represent the residuals obtained during backscatter attenuation estimation with and without segmentation, respectively.

The residuals obtained during the attenuation coefficient estimation fitting process were found to be greater towards the lower end of the usable bandwidth without using the segmentation algorithm (Fig. 8). This was probably due to the relative difference in backscatter coefficient as a function of frequency between the relatively large and flat leek pieces, and the much finer cellulose scatterers. When implementing the segmentation algorithm the frequency dependence of these residuals was largely eliminated for all combinations of ROI window size and phantom positions, potentially validating the use of the mean averaged over frequencies as the test parameter by which to control the algorithm’s feedback loop.

### 4.2 In vivo

**Fig. 9:**
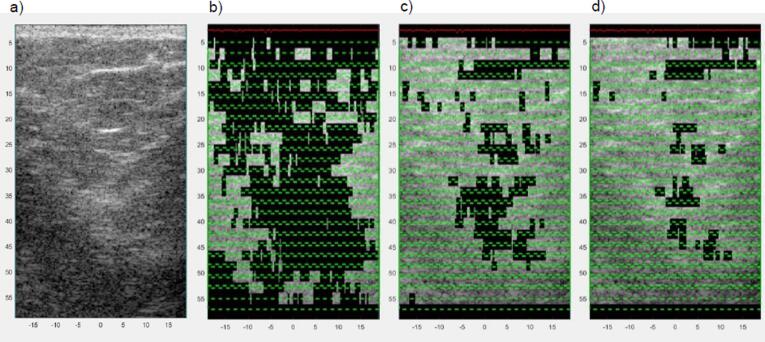
Example of a clinical B-mode image of the breast from volunteer 2. The original B-mode image overlain with a pattern that indicates the ROI windows is shown (a), followed by the image that resulted from segmentation with mean target residual range 5.6 to 6.4 dB (b), 6.0 to 6.8 dB (b), and 6.4 to 7.2 dB (d).

**Fig. 10:**
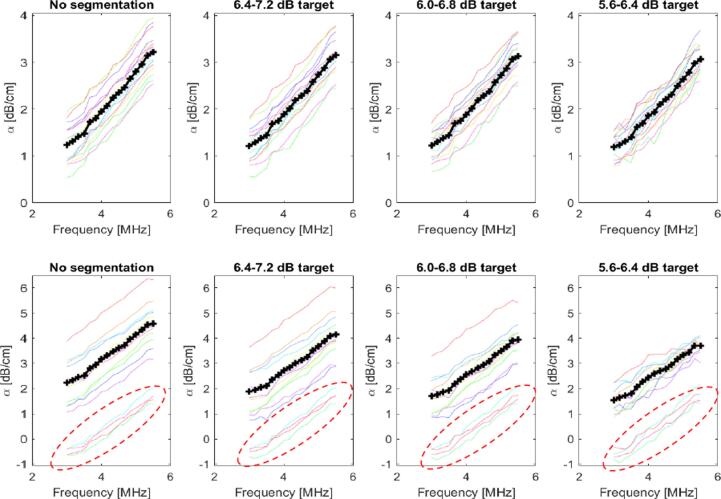
Summary of the attenuation coefficient estimates for volunteer 1 (top row) and volunteer 2 (bottom row) using 6.0 μs ROI window size. Results are divided according to no segmentation/segmentation and, for the latter, target residual range. Coloured lines indicate results from indiviudal images, the black lines with markers (+) indicate the average over results for all images. For volunteer 2 erroenous data with negative coefficients are indicated by the dashed red ellipse.

Fig. 9 provides an example B-mode image of the breast *in vivo* (25 year-old female) and illustrates the effect on the segmentation of varying the mean TRR. In this example the more conservative TRR remove the most apparent hyperechoic image features, while a lower TRR (5.6–6.4 dB) closer to the one used in the phantom study removed a significant percentage (>60%) of the data. Applying the various TRRs to the data acquired from two volunteers provided frequency dependent attenuation coefficient curves as shown in Fig. 10. Inspection of the results demonstrates that both the variance and the mean value of attenuation coefficient are reduced as the TRR is lowered. In the second volunteer four image sets obtained in the upper–outer quadrant of each breast provided low attenuation coefficients including some negative values.

These images were characterised by relatively large proximal regions of hypo-echoic backscatter, and distal regions with relatively higher backscatter. Under these conditions the algorithm was not able to deal with the spatial backscatter distribution without producing low attenuation coefficient estimates. The results from these images are included and indicated in Fig. 10, but not considered when quantifying the attenuation coefficient statistics (Table 3) across images obtained at different positions in this volunteer.

**Table 3:** The mean, standard deviation and range of the attenuation frequency slope (dB/cm/MHz), and the mean residual in the frequency dependence fit. Values were determined across image data sets for volunteer 1 (n = 16) and volunteer 2 (n = 12). For volunteer 2 the erroneous results identified in the text and Fig. 12 were omitted from the computation.

	**Volunteer 1**				**Volunteer 2**			
	**α Freq. Slope (dB/cm/MHz)**			**Freq. Dep. Fit residual (dB/cm)**	**α Freq. Slope (dB/cm/MHz)**			**Freq. Dep. Fit residual (dB/cm)**
	**Mean**	**St. dev.**	**Range**		**Mean**	**St. dev.**	**Range**	
**No segmentation**	0.51	0.10	0.31	0.059	0.80	0.21	0.74	0.078
**6.4**–**7.2 dB Target**	0.50	0.09	0.33	0.059	0.69	0.22	0.76	0.082
**6.0**–**6.8 dB Target**	0.50	0.08	0.28	0.060	0.65	0.17	0.64	0.090
**5.6**–**6.4 dB Target**	0.48	0.07	0.21	0.085	0.62	0.09	0.27	0.128

Table 3 summarises the mean, standard deviation and range of the attenuation coefficient frequency–slope obtained by no segmentation, and by varying the mean TRR. The mean slope was greater for the second volunteer. The range of the slope was reduced from 0.31 to 0.21 dB/cm/MHz, and from 0.74 to 0.21 dB/cm/MHz for volunteer 1 and 2 respectively. The mean attenuation coefficient versus frequency fit residual from each data set is also included in Table 3. These data show how applying the segmentation algorithm with the lower TRR produces noisier attenuation coefficient frequency dependence curves (0.085 vs 0.05 dB/cm, and 0.128 vs. 0.078 dB/cm for volunteers 1 and 2 respectively).

**Fig. 11:**
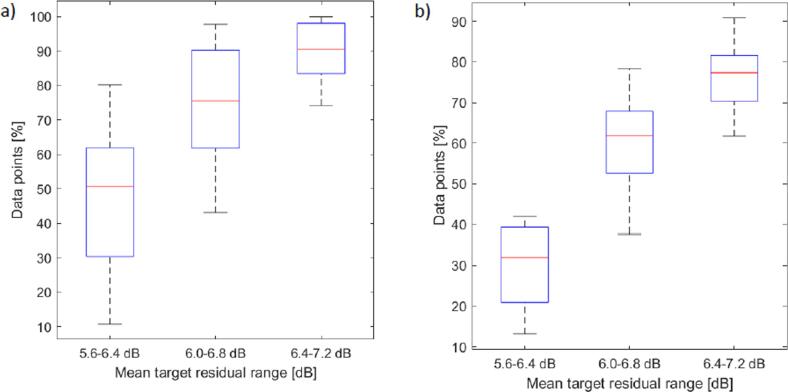
The percentage of the total data used with differing target residual settings is summarised in plots (a) and (b) for volunter 1 and 2 respectively.

Fig. 11 summarises the amount of data used in attenuation estimation, as a percentage of the data used without segmentation applied. As would be expected a higher percentage of data was removed with the lower TRR. Within a specific TRR a wide spread of percentage data values were also observed, indicating that it is not possible to predict exactly how a given TRR will impact the amount of data segmentation. This provides potential evidence of differences in the amount of variance in backscatter across different breasts, as well as within the same breast.

**Fig. 12:**
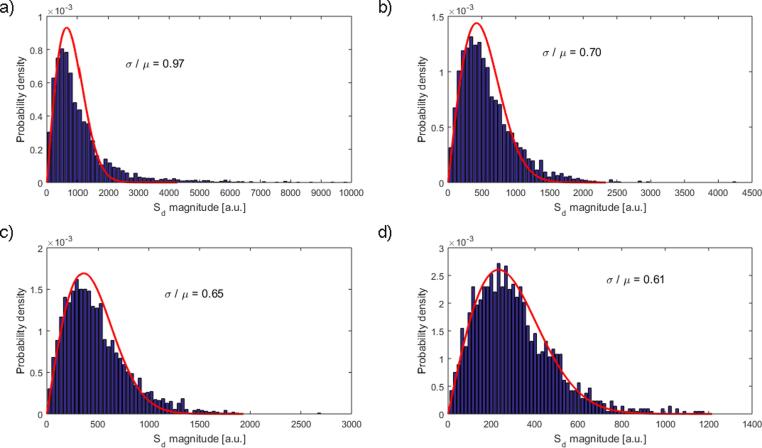
Example probability density functions computed from attenuation corrected S_d_ values from the in-vivo clinical breast data from volunteer 2. Histograms and Rayleigh distribution fit are shown for no segmentation (a), 6.4–7.2 dB TRR (b), 6.0–6.8 dB TRR (c), and 5.6 – 6.4 dB TRR (d) respectively. The standard deviation (*σ*) to mean (*µ*) ratio for each histogram is included.

An analysis of the segmented backscatter data distributions revealed a shift towards a Rayleigh distribution as the TRR was reduced (Fig. 12). The standard deviation to mean ratio for each image and TRR combination was computed, giving mean values of 0.99 ± 0.14, 0.73 ± 0.14, 0.67 ± 0.03 and 0.61 ± 0.02 for no segmentation, TRR of 6.4–7.2, TRR of 6.0–6.8 dB and TRR of 5.6–6.4 dB respectively.

## 5 Discussion

In this work we considered the effect of backscatter inhomogeneity on attenuation coefficient estimates and we introduced an algorithm aimed at segmenting echoes originating from backscatter outliers prior to attenuation coefficient estimation. We proceeded to test the algorithm on a specially designed phantom and on *in vivo* data from the human breast.

A test phantom was designed with carefully positioned backscatter outliers so that backscatter attenuation estimates obtained without segmentation would suffer large errors when compared with independent transmission attenuation measurements. The automated segmentation algorithm was developed and shown to be effective in segmenting out the backscatter outliers, providing attenuation estimates with a large reduction (60–80%) in error when compared to the values obtained when no segmentation was attempted (Fig. 6, Fig. 7, Table 1). In the situation where the backscatter outliers were positioned relatively homogeneously (positions 6 and 7) through the phantom an unsurprising result was that without segmentation the error was not as large as in other positions. These results demonstrate the sensitivity of attenuation measurements to the spatial distribution of the backscatter outliers. Ultimately the benefit of the segmentation algorithm becomes more apparent when there is some form of inhomogeneity in the spatial distribution of the backscatter outliers that can be successfully detected.

The phantom results reported here extend previous work by other authors who considered the effects of backscatter inhomogeneity in attenuation coefficient measurements. Laugier *et al* [30] considered the effect of a specular reflector embedded in an otherwise uniformly backscattering material on attenuation coefficient estimates, demonstrating a bias effect similar to that described here, suggesting removal or substitution of the echo data originating from the specular reflector prior to attenuation coefficient estimation. Nam *et al* [21] considered the problem of backscatter and attenuation inhomogeneity and proposed a reference method [14] The main difference between our results and those by Nam *et al* [21] are that we have considered phantoms with multiple, relatively thin, reflectors. Furthermore, our algorithm actively aims to detect and segment out the echoes originating from such features.

The segmentation algorithm was implemented in a way that requires a characterisation of the diffraction field and an initial estimate of the attenuation frequency dependence. For clinical use where a result is needed from individual images, a practical solution is to characterise the diffraction field a-priori perhaps with a tissue-mimicking phantom or other, in a manner similar to the approach taken here with the *in vivo* breast image data. Since characterisation of the diffraction field is a requirement for attenuation estimation, the same data set can be used to help determine the segmentation threshold function. A potential problem here is that the properties of the diffraction calibration phantom may not be suitable for the scattering properties of the *in vivo* tissue of interest. The validity of such an approach is of interest but is beyond the scope of this particular study. The initial estimate of attenuation frequency dependence required by the segmentation algorithm is a potential source of error. The segmentation algorithm works by adjusting the attenuation frequency dependence parameter at each iteration of the threshold function. Provided the initial estimate of the attenuation frequency dependence is accurate, within an order of magnitude perhaps, the algorithm would be expected to quickly converge towards a correct value, as was found in both the phantom and *in vivo* data. The choice of the 1/3 factor in adjusting the scaling parameter *s* in the automated algorithm was arbitrary. This value provided an efficient means for the algorithm to converge towards an answer, however there may be situations where larger or smaller adjustment factors might be required depending on whether a faster or more precise algorithm is needed.

An inevitable consequence of segmentation is the reduction in the backscatter data available for attenuation estimation. This reduction in data can be tolerated provided the removal of backscatter outliers effectively leads towards more accurate answers. If the degree of segmentation is large, accuracy may be improved but at the expense of precision, where the final result may not offer any effective benefit over more precise but inaccurate estimates obtained without any attempt at segmentation. In cases where there are many structures to be segmented out a problem can be posed by the relative distance between these structures and the length of the ROI window. In this study the criteria for data segmentation was that the segmentation mask produced by applying the threshold function should not overlap with the central half of the ROI windows. This criterion was chosen on the basis that any high amplitude echo towards the edges of the ROI window would be suppressed by the use of Hanning windowing. It follows that the use of a large ROI window size will lead to a larger percentage removal of the available data for backscatter attenuation estimation particularly if there are many closely spaced backscatter outliers. In such a situation the choice of a smaller ROI size may be preferable as it is more likely to preserve data in the gaps between the backscatter outliers at the expense of reduced frequency resolution. In the phantom study the use of a short (12 cycles) ROI window size did not present any adverse effect on the accuracy of the results when compared to the larger window sizes. In cases where high frequency resolution is not considered critical, the use of a small ROI would therefore be recommended in order to retain as much of the backscatter data as possible.

Backscatter attenuation estimates from the *in vivo* breast data (Fig. 10) provided a greater challenge than the phantom which was constructed specifically to test the segmentation algorithm. Inhomogeneity in backscatter and attenuation coefficients *in vivo* is likely to be complex and dependent on the target tissue type and any tissue pathology. Detecting backscatter outliers *in vivo* was more challenging and setting an appropriate threshold scaling value in the *in vivo* images was not as straightforward as in the phantom. In this study we found that using a low TRR (5.6–6.4 dB) similar to the phantom study could lead to a large reduction in the available data for attenuation estimation, therefore *in vivo* it may be necessary to maintain a slightly larger TRR (6.0 to 6.8 dB) to obtain satisfactory results. These results could be considered consistent with the findings of other researchers who investigated RF envelope statistics [24], [25] who correlated changes in the envelope signal to noise ratio to variance in the scattering properties in tissue. In this study we only considered image data from two healthy volunteers with tissue thickness large enough (>4 cm) to provide sufficient data for more precise attenuation coefficient estimates. We are therefore unable to make a suitable recommendation on the TRR that should be used based on these data alone. A further study across many subjects would be required to investigate this aspect more thoroughly, considering factors such as age, tissue pathology and tissue thickness, in order to provide a more definite answer. Another interesting aspect worth investigation would be to determine whether probability density functions other than Rayleigh (homodyned-K, Rice, Nakagami) would be a more suitable choice to model echoes from in to vivo tissue. Indeed, more complex distributions, such as the homodyned-k distribution for example, may allow a hybrid segmentation method based on both intensity and RF envelope statistics.

Unlike the phantom, it was not possible to verify the *in vivo* attenuation coefficients with an independent measurement. The data reported here (Fig. 10, Fig. 11) does however show a reduction in the variance of attenuation coefficient estimates across different breast positions when applying the segmentation algorithm for both volunteer data sets. If it can be assumed that the mean attenuation does not vary within a single breast, this reduction in variance could be taken as evidence of improved precision of estimates using the segmentation algorithm. An increase in the mean residual from the frequency dependence fitting process was also observed when applying the segmentation algorithm. This finding appears to be at odds with the reduction in variance of attenuation measurements across different positions. This discrepancy could however be understood in terms of the sources of uncertainty in the attenuation measurement. Uncertainty associated with the backscatter speckle statistics will be exacerbated when the number of available RF echoes to be averaged is reduced, such as when the segmentation algorithm is applied. On the other hand backscatter outliers can be distributed randomly from one image to another, and depending on their position a positive or negative bias may be introduced as demonstrated in the phantom study. Hence the effect of the segmentation algorithm can be understood in terms of increased accuracy due to segmentation of backscatter outliers (reduction in variance across images), at the expense of precision due to a reduction in the amount of backscatter data that can be averaged.

In this study one assumption was that the structures to be segmented out are those providing higher echogenicity than the surroundings. This assumption may hold for some *in vivo* images, for example where the outliers arise from boundaries between different types of tissue generating reflections with greater intensity than the background homogeneous backscatter. The breast images shown provided good examples of this situation and the phantom with the leek pieces was designed to crudely mimic it. There will be however occasions where it may be preferable to segment out hypoechoic regions, for example cysts. In principle the segmentation algorithm proposed here could be adapted to function in a similar way but with the threshold function segmenting out features which fall below the function rather than above it. Combining two threshold functions may be possible, for example an upper threshold function to be adjusted automatically and a lower threshold function to be set manually. Alternatively, the algorithm presented here could be developed further to automatically adjust scaling factors for both the upper and lower threshold functions. Another approach altogether would be to perform an iterative outlier removal process directly on the diffraction corrected backscatter data, without the need to define a threshold function.

## 6 Conclusion

A novel automatic echo-amplitude based segmentation algorithm has been developed, aimed at improving the accuracy and precision of attenuation coefficient estimates obtained from analysing radiofrequency echoes in medical ultrasound imaging. In tests on a phantom which included backscatter outliers the segmentation algorithm produced attenuation coefficient estimates which improved the agreement with an independent transmission-based method, improving accuracy by up to 80% and precision by 90% compared to results obtained with no attempt at segmentation. Tests on clinical B-mode radiofrequency data obtained from female breasts showed that the algorithm has potential for *in vivo* use, a 5–25% significant reduction in mean attenuation coefficient estimates and a 30–50% significant reduction in standard deviation of attenuation coefficient across different positions within each breast was found. Specific tests are now needed on different target organs to verify the full impact of the automated segmentation algorithm on backscatter attenuation estimates.

## Declaration of Competing Interest

The author declare that there is no conflict of interest.

## References

[1] Kuc R., Schwarz M. (1979). Estimating the acoustic attenuation coefficient slope for liver from reflected ultrasound signals. IEEE Trans Sonics Ultrason..

[2] Bamber J.C., Hill C.R. (1981). Acoustic properties of normal and cancerous human liver—I. Dependence on pathological condition. Ultras Med. Biol..

[3] Landini L., Sarnelli R. (1986). Evaluation of the attenuation coefficients in normal and pathological breast tissue. Med. Biol. Eng. Compu..

[4] D’Astous F.T., Foster F.S. (1986). Frequency dependence of ultrasound attenuation and backscatter in breast tissue. Ultras. In Med. Biol..

[5] Nam K., Zagzebski J.A., Hall T.J. (2013). Quantitative assessment of in vivo breast masses using ultrasound attenuation and backscatter. Ultrason. Imag..

[6] Fujii Y., Taniguchi N., Itoh K., Omoto K. (2003). Attenuation coefficient measurement in the thyroid. J. Ultrasound Med..

[7] Kiss M.Z., Varghese T.M., Kliewer M.A. (2011). Ex vivo ultrasoundattenuation coefficient for human cervical and uterine tissue from 5 to 10 MHz. Ultrasonics.

[8] Ophir J., Maklad N.F., Bigelow R.H. (1982). Ultrasonic attenuation measurements of in vivo human muscle. Ultrason. Imag..

[9] Duric N., Littrup P., Poulo L., Babkin A., Pevzner R., Holsapple E., Rama O., Glide C. (2007). Detection of breast cancer with ultrasound tomography: first results with the Computed Ultrasound Risk Evaluation (CURE) prototype. Med Phys..

[10] O'Flynn E., Fromageau J., Ledger A., Messa A., D'Aquino A., Schoemaker M., Schmidt M., Duric N., Swerdlow A., Bamber J. (2017). Ultrasound tomography evaluation of breast density a comparison with noncontrast magnetic resonance imaging. Invest Radiol..

[11] Cloostermans M.J., Thijssen J.M. (1983). A beam corrected estimation of the frequency dependent attenuation of biological tissues from backscattered ultrasound. Ultrasonics Imaging.

[12] Parker K.J., Lerner R.M., Waag R.C. (1984). Attenuation of ultrasound: magnitude and frequency dependence for tissue characterization. Radiology.

[13] Laugier P., Berger G., Fink M., Perrin J. (1987). Diffraction correction for focused transducers in attenuation measurements in vivo. Ultrason. Imaging.

[14] Yao L.X., Zagzebski J.A., Madsen E.L. (1990). Backscatter coefficient measurements using a reference phantom to extract depth-dependent instrumentation factors. Ultrason Imaging.

[15] Kuc R. (1984). Estimating acoustic attenuation from reflected ultrasound signals: Comparison of spectral-shift and spectral-difference approaches. IEEE Trans. Acoust. Speech Signal Process..

[16] Fink M.A., Cardoso J.F. (1984). Diffraction effects in pulse-echo measurement. IEEE Trans. Sonics Ultras..

[17] Abbott J.G., Thurstone F.L. (1979). Acoustic speckle: theory and experimental analysis. Ultrason. Imaging.

[18] Parker K.J. (1986). Attenuation measurement uncertainties caused by speckle statistics. J. Acoust. Soc. Am..

[19] Kuc R., Schwartz M., Von Micsky L. (1976). Parametric estimation of the acoustic attenuation coefficient slope for soft tissue.

[20] Labyed Y., Bigelow T.A. (2011). A theoretical comparison of attenuation measurement techniques from backscattered ultrasound echoes. J. Acoust. Soc. Am..

[21] Nam K., Zagzebski J.A., Hall T.J. (2011). Simultaneous backscatter and attenuation estimation using a least squares method with constraints. Ultrasound. Med. Biol..

[22] Tu H., Varghese T., Madsen E.L., Chen Q., Zagzebski J.A. (2003). Ultrasound attenuation imaging using compound acquisition and processing. Ultrason. Imaging.

[23] Zenteno O, Luchies A, Oelze A, Lavarello R. Improving the quality of attenuation imaging using full angular spatial compounding, in: Proc. IEEE Ultrason. Symp. 2014:2426-2429.

[24] Rubert N., Varghese T. (2014). Scatterer number density considerations in reference phantom-based attenuation estimation. Ultrasound Med. Biol..

[25] Deeba F., Ma M., Pesteie M., Terry J., Pugash D., Hutcheon J.A., Mayer C., Salcudean S., Rohling R. (2019). Attenuation coefficient estimation of normal placentas. Ultrasound Med. Biol..

[26] Coila A.L., Lavarello R. (2017). Regularized spectral log difference technique for ultrasonic attenuation imaging. IEEE. Trans. Sonics Ultrason..

[27] Deeba F., Hu R., Terry J., Pugash D., Hutcheon J.A., Mayer C., Salcudean S., Rohling R. (2019). A spatially weighted regularization method for attenuation coefficient estimation. IEEE International Ultrasonics Symposium (IUS), Glasgow, United Kingdom.

[28] F. Destrempes, M. Gesnik, G. Cloutier, Construction of adaptively regularized parametric maps for quantitative ultrasound imaging, IEEE International Ultrasonics Symposium (IUS), Glasgow, United Kingdom, 2019, pp. 2027-2030.

[29] Romero SE, Coila A, Lavarello RJ. A regularized quantitative ultrasound method for simultaneous calculation of backscatter and attenuation coefficients. Proc. SPIE 11330, 15th International Symposium on Medical Information Processing and Analysis, 113300T, 2020.

[30] Laugier P., Berger G., Fink M., Perrin J. (1985). Specular reflector noise: effect and correction for in vivo attenuation estimation. Ultrason. Imaging.

